# Bremelanotide for Treatment of Female Hypoactive Sexual Desire

**DOI:** 10.3390/neurolint14010006

**Published:** 2022-01-04

**Authors:** Amber N. Edinoff, Nicole M. Sanders, Kyle B. Lewis, Tucker L. Apgar, Elyse M. Cornett, Adam M. Kaye, Alan D. Kaye

**Affiliations:** 1 Department of Psychiatry and Behavioral Medicine, Louisiana State University Health Science Center Shreveport, Shreveport, LA 71103, USA; 2 Shreveport School of Medicine, Louisiana State University, Shreveport, LA 71103, USA; nms002@lsuhs.edu (N.M.S.); kbl004@lsuhs.edu (K.B.L.); 3 Department of Chemical Biology and Biochemistry, Vanderbilt University, Nashville, TN 37235, USA; tuckerapgar22@gmail.com; 4 Department of Anesthesiology, Louisiana State University Health Science Center Shreveport, Shreveport, LA 71103, USA; elyse.bradley@lsuhs.edu (E.M.C.); alan.kaye@lsuhs.edu (A.D.K.); 5 Department of Pharmacy Practice, Thomas J. Long School of Pharmacy and Health Sciences, University of the Pacific, Stockton, CA 95211, USA; akaye@pacific.edu

**Keywords:** hypoactive sexual desire disorder, bremelanotide, melanocortin receptor agonist

## Abstract

Hypoactive sexual desire disorder (HSDD) is a persistent deficiency or absence of sexual fantasies and desire resulting in significant distress or interpersonal difficulty. Women with this disorder may display a lack of motivation for sexual activity, reduced responsiveness to erotic cues, a loss of interest during sexual activity, and avoidance of situations that could lead to sexual activity. The pathophysiology of HSDD is thought to be centered around inhibitory and excitatory hormones, neurotransmitters, and specific brain anatomy. Due to the multifactorial nature of HSDD, treatment can be complex and must attempt to target the biological and psychosocial aspects of the disorder. Bremelanotide is a melanocortin receptor agonist and has been recently approved by the FDA to treat HSDD. Bremelanotide is administered intranasally or as a subcutaneous injection. The recommended dosage of bremelanotide is 1.75 mg injected subcutaneously in the abdomen or thigh at least 45 min before sexual activity. Studies showed improvements in desire, arousal, and orgasm scores when 1.75 mg of bremelanotide was administered before sexual activity compared to a placebo. Bremelanotide is a promising way to treat HSDD.

## 1. Introduction

Hypoactive sexual desire disorder (HSDD) is the most prevalent female sexual health disorder [1]. HSDD is defined as persistent deficiency or absence of sexual fantasies and desire, resulting in significant distress or interpersonal difficulty [2]. The criteria based on the Diagnostic and Statistical manual are “persistently or recurrently deficient (or absent) sexual fantasies and desire for sexual activity: that causes marked distress or interpersonal difficulty” [3]. Women with this disorder may display a lack of motivation for sexual activity, reduced responsiveness to erotic cues, a loss of interest during sexual activity, and avoidance of situations that could lead to sexual activity. Women who suffer from HSDD commonly have reduced self-confidence, self-worth, and impaired body image [2]. Diagnosis of HSDD requires personal distress and a change in sexual desire for a least three months [4,5]. Subtypes of HSDD include those that are due solely to “psychological factors” and “combined factors.” The HSDD can be due to psychological factors, substances, or medical conditions in the combined subtype. The overall prevalence of HSDD ranges from 8–19% [1,6,7,8]. The prevalence of HSDD increases with age and has a higher prevalence in postmenopausal women. The risk is the greatest in younger women in surgical menopause and is associated with a less active sex life and decreased sexual and relationship satisfaction [1]. Women who are premenopausal and experience hypoactive sexual desire have higher levels of distress than postmenopausal women and are more likely to seek help [9]. Women with distressing low sexual desire have a poorer health-related quality of life. Women suffering from this disorder have reported decreased physical functioning, vitality, social functioning, general health, mental health, and a loss of femininity [1,10].

Several factors have been proposed to explain the etiology of low sexual desire and the accompanying distress. These factors include partners and life situation, ethnicity and culture, menopause status, central nervous system activity, and hormonal influences [10]. Married women or women living with a spouse or partner were more likely to have distressing low sexual desire than single women [11]. The PRESIDE study revealed that Caucasian women were more likely than Black women to have low sexual desire [10]. Additionally, brain areas such as the cerebral cortex may play a role in women developing HSDD. When presented with erotic cues, women with HSDD were shown to have weaker cerebral cortex activation in the right hemisphere and less deactivation in the left hemisphere than women who did not have HSDD [12]. Various drugs or conditions that decrease dopamine levels alter brain serotonin at serotonin 2A receptors or increase opioids at mu receptors have also been shown to reduce or inhibit sexual desire [13,14]. Treatment of HSDD includes both psychosocial and biological therapies. Psychosocial therapies include cognitive behavioral therapy, sensate focus therapy, and mindfulness-based cognitive therapy. The current biological therapies for HSDD include flibanserin, testosterone therapy, bupropion (off-label treatment), and buspirone (off-label treatment) [2].

## 2. Hypoactive Sexual Desire

### 2.1. Risk Factors

HSDD often goes underdiagnosed and undertreated due to the private nature of the condition, making it difficult for patients to discuss with physicians. Understanding and identifying risk factors associated with HSDD may help medical providers initiate conversations with patients. Several factors have been associated with a potentially increased risk of HSDD, including biological, psychosocial, and pharmacological influences. Some commonly studied biological risk factors for HSDD include age, hormone level, and other comorbidities [15,16,17]. In a study on sexual distress in US women, Shifren et al. noted that although sexual dysfunction increases with age, actual personal distress about sexual issues decreases with age. This study reported that sexual distress occurred in 12% of the studied population and was more common in mid-aged (45–64 years) women (14.8%), followed by younger women (10.8%) and older women (8.9%) [8]. Other studies of both US and European women have shown that postmenopausal women and women who have undergone oophorectomy (surgical menopause) have lower sexual desire and a higher risk of developing HSDD than pre-menopausal women with younger surgical postmenopausal women being at greatest risk [18]. This finding suggests that the decrease in estrogen and androgens after either natural or surgical menopause plays a role in losing sexual desire and developing HSDD [17,19]. However, other postmenopausal symptoms, such as vaginal dryness, may also lead to low sexual desire, highlighting the importance of excluding other possible causes when considering the diagnosis of HSDD [20]. Comorbidities that have also been associated with HSDD include chronic medical conditions (diabetes, coronary artery disease, etc.), urinary incontinence, multiple sclerosis, Parkinson’s disease, and head injury [17]. Leiblum et al. reported that women with HSDD had significantly more general health issues than women without HSDD [1].

Studies have also reported psychosocial risks related to HSDD, including depression, relationship status, and culture. Depression can both lead to and be a result of HSDD, which is why it is important to determine the onset of depressive symptoms in relation to low sexual desire and personal distress [10]. A study by Whalin-Jacobsen et al. looked at the association of androgen levels and psychosocial factors with HSDD and found that androgen levels were associated with low sexual desire but not with HSDD [21]. However, relationship length and depressive symptoms were positively associated with HSDD, underlining the importance of using a biopsychosocial model to understand and diagnose HSDD [12]. The prevalence of low sexual desire has been reported to be similar among surgically postmenopausal women of European descent; however, HSDD was more prevalent in France (22%) than in Germany (7%), which suggests that culture plays a role in developing HSDD [15].

Finally, it is important to consider pharmacological risks eliciting HSDD symptoms. Selective Serotonin Reuptake Inhibitors (SSRIs) cause an increase in serotonin, which is considered an inhibitory signal of the sexual drive leading to a blunted sexual response and distressing low sexual desire. Opioid consumption can also be a cause of hypoactive sexual desire [22]. Opioids can inhibit ovarian sex hormones and adrenal androgen production, which was found in a study that looked at women who were chronically using sustained-action opioids [22,23]. Opioids exert their effects on adrenal androgen production through their inhibitory effects on the hypothalamo-pituitary-adrenal axis [23]. However, pharmacologically induced HSDD does not fit the *Diagnostic Statistical Manual* IV (DSM-IV) criteria of HSDD.

### 2.2. Pathophysiology

The pathophysiology of HSDD is thought to be centered around inhibitory and excitatory hormones, neurotransmitters, and specific brain anatomy [16,24,25]. Estrogen, testosterone, progesterone, and dopamine positively affect sexual desire, whereas serotonin, opioids, and prolactin negatively affect sexual desire. Studies on rats suggest that estrogen and testosterone exert their excitatory effect on sexual response via increasing dopamine release and synthesis, respectively [26,27]. Conversely, serotonin reduces dopamine’s positive effect on sexual response, leading to its inhibitory effect [25].

There is support in animal studies that testosterone also has a role in sexual desire. A study performed by Maseroli et al. looked at non-aromatized androgen dihydrotestoerone (DHT) and sexual behavior in female rats. The rats who were primed with estrogen and received DHT displayed significantly more appetitive behaviors compared to the negative controls [28]. The authors concluded that the administration of DHT enhances sexual behavior. This argues that testosterone does play a role in sexual desire, suggesting that it may also play a role in hypoactive sexual desire.

Neuroimaging studies have shown that HSDD is related to a sexual desire brain network (SDBN) involving different areas of the brain that are either excited or repressed by sexual stimuli. These studies suggest that atrophy of excitatory areas and hyperactivity in inhibitory areas may lead to an increased risk of HSDD [24]. This study also reports that this SDBN upholds the top-down processing model of HSDD, suggesting that women with HSDD focus their attention more on evaluating their response to sexual stimuli rather than on allowing themselves to be stimulated.

## 3. Current Treatment

Since a variety of biological, psychological, and social factors cause HSDD, current treatments follow a biopsychosocial approach when evaluating and treating patients affected by the disorder [17,29,30]. Literature sources agree that there are significant challenges in the treatment of HSDD due to the lack of structured treatment regimens and clear clinical guidelines, so treatments may vary on an individualized basis. Recommended treatments start with office-based counseling before progressing to psychotherapy and/or pharmacotherapy [17]. Prior to discussing the available treatment options, it is important to note that approved treatments are limited in women with HSDD who are postmenopausal. In this section, the current treatment will be discussed.

### 3.1. Office-Based Counseling

Initially, office-based counseling may help treat HSDD with basic education and recommended lifestyle changes to improve sexual desire [31]. Clinician reluctance to discuss sexual health is a significant barrier for treating patients with HSDD. As a result, clinicians must be proactive in identifying sexual concerns and determining the best available treatment options. One popular approach for office-based counseling is the PLISSIT model, an approach used for the treatment of sexual disorders. The model incorporates permission to discuss problems and emotions, limited information on basic sexual function education and resources, specific suggestions to address problems with directives, and the need for more intensive treatment [32].

### 3.2. Psychological

Subsequently, psychological intervention has been recommended as a next treatment modality if office-based counseling is ineffective or severe psychiatric issues, such as trauma or abuse, are revealed for individual cases. Studies have suggested a collection of separate interventions for female sexual dysfunction include cognitive-behavioral therapy (CBT), mindfulness meditation therapy (MMT), and exercises for couples. However, although controlled trials support the use of CBT and MMT for the treatment of HSDD, the efficacy of these interventions has not been supported by scientific and regulatory standards for drug treatment trials. Specifically, trials testing the efficacy of psychological treatments lack the scientific and regulatory standards necessary for significant results. Standards include reproducibility of intervention, randomizations, adequate control, and/or outcomes of clinical relevance [33]

### 3.3. Pharmacological

A variety of pharmacological treatments have been tested for HSDD through randomized controlled clinical trials. However, current treatment options approved by the FDA are still limited for women with the disorder. Treatment options focus on the inhibitory and excitatory pathways linked to the regulation of responses for sexual cues. There are ongoing investigations for novel treatments against HSDD cover hormone therapies and centrally acting drugs intended to regulate these neural pathways [31]. Studies have measured efficacy using patient-reported outcomes of sexual desire [34,35].

### 3.4. Off-Label Treatments

Alternative pharmacological approaches have been studied using off-label medications. One of the most common treatments tested for HSDD has been testosterone. Testosterone is the primary sex hormone associated with the regulation of sexual desire, and low levels in postmenopausal women are associated with loss of libido and decreased sexual activity [17]. HSDD has been hypothesized to occur due to low circulating androgen levels arising from either post-menopause or surgical removal of the ovaries [36,37]. Currently, data support that testosterone treatment shows efficacy in women with low levels and a decrease in sexual desire [38,39,40,41,42,43]. Likewise, combination therapy of estrogen and methyltestosterone has been shown to be a viable treatment for HSDD. However, studies evaluating testosterone treatment for HSDD have not evaluated long-term treatments nor have they established detailed results on safety and tolerability [44]. Currently, treatments incorporating testosterone with other medications such as sildenafil are being evaluated for improved sexual functioning [45].

Another off-label treatment being tested for HSDD is bupropion. Bupropion, a dopamine and serotonin reuptake inhibitor, shows a lower incidence of sexual dysfunction in patients with major depressive disorder [46,47,48,49,50]. In studies evaluating women with HSDD, bupropion had a significantly increased rate of release compared to placebo [51,52]. It is thought that the increased availability of dopamine is what helps to decrease the risk of sexual dysfunction and could be the reason it may be helpful in treating HSDD.

### 3.5. Approved Agents

Flibanserin (postsynaptic 5-hydroxytryptamine 1A agonist and 2A antagonist) was the first agent approved by the FDA for the treatment of HSDD [53]. Flibanserin functions by decreasing serotonin levels and increasing both dopamine and norepinephrine levels [13,54]. Flibanserin has a high affinity for 5-HT_1A_ receptors in the hippocampus and the prefrontal cortex [55]. It also has agonist activity at the 5-HT_1A_ postsynaptic receptors, which has some downstream effects in altering the levels of other monoamines, including dopamine [55]. Flibanserin also causes a net increase in norepinephrine concentrations in the prefrontal cortex through the disinhibition of the locus coeruleus noradrenergic neurons [55]. These neurotransmitters have been associated with excitatory and inhibitory responses to sexual cues implicated in HSDD [13]. Clinical trials have demonstrated that subjects experienced improved satisfying sexual events after taking flibanserin compared to that of the control group [56,57,58,59,60,61,62,63,64]. Flibanserin was initially rejected for approval by the FDA in 2010 due to concerns over efficacy and safety data [53,65]. The most common adverse events included somnolence and dizziness, which researchers have explored with alcohol use [54,62]. Finally, Bremelanotide, which is the main focus of this paper, is a melanocortin receptor agonist and has been recently approved by the FDA for the treatment of HSDD [10,66]

## 4. Bremelanotide

Bremelanotide is a melanocortin receptor agonist which non-selectively activates melanocortin 1 receptor (MC1R), melanocortin 2 receptor (MC2R), melanocortin 3 receptor (MC3R), melanocortin 4 receptor (MC4R), and melanocortin 5 receptor (MC5R) receptors. Activation of MC4R receptors modifies brain pathways involved in sexual responses [67]. Stimulation of MC4R receptors can also cause a transient increase in blood pressure and a decrease in heart rate [68]. Bremelanotide is contraindicated in individuals with uncontrolled hypertension or cardiovascular disease [69]. Activation of MC1R receptors may contribute to the adverse effect of hyperpigmentation [67]. Bremelanotide is able to be administered intranasally or as a subcutaneous injection. The subcutaneous route has 100% bioavailability and is associated with fewer side effects [68]. The recommended dosage of bremelanotide is 1.75 mg injected subcutaneously in the abdomen or thigh at least 45 min prior to sexual activity. Individuals should take only one dose every 24 h. Individuals should refrain from taking more than eight doses of bremelanotide per month.

### 4.1. Mechanism of Action

The mechanism of action of bremelanotide is better understood for treating male sexual dysfunction compared to female sexual dysfunction. In males, bremelanotide primarily acts on MC3R and MC4R to help treat erectile dysfunction. The stimulation of the melanocortin receptors, in general, causes a local increase of nitric oxide in the penis leading to vasodilation and penile erection [70,71]. In females who suffer from HSDD, abnormal sexual responses are due to an imbalance of various neurotransmitters. Amongst these neurotransmitters, dopamine, and melanocortin stimulate attention and desire while norepinephrine and oxytocin stimulate sexual arousal [72]. In females, administration of bremelanotide acts primarily on the presynaptic MC4R and stimulates the release of dopamine to portions of the nucleus accumbens, medial preoptic area, verbal tegmental area, arcuate nucleus, and the medial and basolateral amygdala [66]. These brain areas are involved in regulating the motivational, arousal, and appetitive aspects of sexual behavior [73].

### 4.2. Pharmacodynamics of Bremelanotide

Bremelanotide is a non-selective agonist of the melanocortin receptors but is thought to mainly act as an MC3R and MC4R receptor agonist. It is important to note that agonism of the melanocortin receptors may lead to increased melanin expression, which can lead to hyperpigmentation [74]. The main adverse effects of bremelanotide include transient increases in systolic and diastolic blood pressure, nausea, headache, and hyperpigmentation [75]. In a study addressing the pharmacodynamics of bremelanotide, researchers monitored ambulatory blood pressures of premenopausal women who received the drug daily for eight days. They found a mean increase of 1.9 mmHg (daily SBP) and 1.7 mmHg (daily DBP). Elevated SBP and DBP measurements peaked at 2.8 mmHg 4–8 h after receiving a dose of bremelanotide and at 2.7 mmHg 0–4 h after receiving a dose, respectively.

### 4.3. Pharmacokinetics of Bremelanotide

After subcutaneous administration of bremelanotide, its mean maximum plasma concentration reaches 72.8 ng/mL, and AUC is 276 hr*ng/mL. Bremelanotide’s Cmax level reaches its plateau after a 7.5 mg dose administration. It takes about 1 h for Bremelanotide to reach its maximum plasma concentration and does have 100% bioavailability with a subcutaneous injection. It is noted to be 21% protein-bound in the serum [74]. After a subcutaneous dose of bremelanotide, its half-life is 2.5 h and has a mean clearance of 6.5 +/− 1.0 L/h. Bremelanotide is a 7 amino acid chain and its metabolism consists of multiple hydrolysis reactions [74]. It is renally excreted (64.8%), with some fecal excretion (22.8%). Renal and hepatic impairment causes an increase in bremelanotide’s AUC. Bremelanotide decreases gastric emptying and has been shown to decrease the rate and extent of absorption of other orally administered drugs, particularly indomethacin and naltrexone. If patients are taking these drugs, they should avoid taking bremelanotide [75].

## 5. Clinical Studies: Safety and Efficacy

Bremelanotide (PT-141) is a melanocortinergic agent, a group of synthetic analogs with demonstrated efficacy in improving male and female sexual dysfunction [66]. Across multiple controlled clinical trials, bremelanotide has been demonstrated to be a possible treatment option for women with hypoactive sexual desire disorder (HSDD).

### 5.1. Early Studies

The earliest clinical study of bremelanotide took place in a 2006 randomized controlled clinical trial that evaluated physiological and subjective measurements of sexual arousal and desire in premenopausal women diagnosed with HSDD [76]. Eighteen women received intranasal doses of bremelanotide or matching placebo on the first and second in-clinic visit. In each session, women were shown a 20-min normal video followed by a 20-min sexually explicit video. The perceived sexual response was evaluated through questionnaires and vaginal vasocongestion, which was measured by vaginal photoplethysmography. When administered bremelanotide compared to the placebo, women reported moderate or high sexual desire (*p* = 0.0114). Women also reported more positive responses to feelings of genital arousal (*p* = 0.0833) and more satisfaction with the level of sexual arousal when attempting sexual intercourse in the following 24 h (*p* = 0.0256). The study reported no adverse effects from bremelanotide administration.

### 5.2. Phase I

To evaluate the safety and tolerability of bremelanotide, a phase I randomized double-blind study evaluated the administration of bremelanotide in conjunction with ethanol for analysis of pharmacokinetic interactions [77]. Patients (*n* = 24, 12 male and 12 female) were randomized to 1 of 6 treatment groups and received single doses of either 20 mg intranasal bremelanotide or placebo on days 1, 4, and 7 over the course of 7 days. Doses were administered with or without 0.6 g/kg ethanol. Adverse events were evaluated using vital signs, self-rated sedation scores, nursing and medical observations, and spontaneous reporting by participants. Blood and urine samples were collected for clinical safety laboratory tests. A physical exam and resting 12-lead electrocardiogram were performed on subjects at baseline and on the 7th study day. In total, the percentage of adverse events was highest for subjects who received ethanol and bremelanotide (75%) compared to subjects who received bremelanotide only (67%) or ethanol only (58%). The study concluded that there were no significant increases in the incidence of adverse events. Small decreases in mean blood pressure were only observed for bremelanotide and ethanol and ethanol alone. There were also no clinically significant changes in blood pressure and no noticeable pharmacokinetic interactions with bremelanotide administration alongside ethanol.

### 5.3. Phase 2b

A double-blind controlled trial in 2016 evaluated the long-term efficacy/safety of bremelanotide administered subcutaneously for premenopausal women with HSDD and/or female sexual arousal disorder (FSAD) [78,79]. Subjects were at least 21 years old, had at least two years of normal sexual function, a stable monogamous relationship for at least six months, and no underlying medical conditions which affected sexual function. Subjects were double-blind placebo groups with either 0.75, 1.25, 1.75 mg BMT, or placebo in a 1:1:1:1 ratio. The groups were stratified by diagnosis (FSAD, HSDD, or both). Initially, two doses were administered one week apart in the clinic before 12 weeks of self-administered doses at home. Subjects would self-administer treatment with subcutaneous injection approximately 45 min prior to sexual activity. Subjects who provided data after four weeks of double-blind treatment were a part of the mITT population (*n* = 327). Primary efficacy endpoints measured the change in sexually satisfying events (SSEs) from baseline to end of the study. Secondary endpoints measured the changes in sexual desire and scores. Desire was scored using the Female Sexual Function Index (FSFI) assesses female sexual function using a self-assessment of sexual feelings and responses on a five- or six-choice scale [34,80]. To assess drug safety, the study used physical examination, vital signs, 12-lead ECGs, and clinical lab tests to evaluate subjects. Blood pressure (BP) was also monitored using 2 h and 24-h ambulatory blood pressure monitoring (ABPM). Patients were excluded from the study if they sustained elevated BP values (SBP ≥ 150–170 mmHg/DBP ≥ 95–105 mmHg) or had a change in baseline (SBP ≥ 30 mmHg/DBP ≥ 15 mmHg) for two to four consecutive readings. This measure was included in response to safety concerns associated with earlier studies of intranasal bremelanotide treatment. Specifically, melanocortin receptor agonists, such as bremelanotide, may cause increases in BP [68].

Overall, 1.25 mg and 1.75 mg bremelanotide were demonstrated to have the greatest efficacy profile. Subjects treated with bremelanotide in the 1.25/1.75 mg groups showed statistically significant improvement in primary and secondary endpoint measures compared to the placebo group. The mean change in number of SSEs was +0.7 (2.4) events/month for bremelanotide compared with +0.2 (2.3) for placebo (*p* = 0.0180). For secondary endpoints, the mean change in FSFI score was +3.6 (5.7) versus +1.9 (5.9; *p* = 0.0017) and the mean change in FSDS-DAO score was −11.1 (12.0) versus −6.8 (13.6; *p* = 0.0014). One specific distinction between the two dosage groups was that 1.75 mg subjects showed a statistically significant increase in all FSDS-DAO and FSFI outcomes compared to the total score and desire score for the 1.25/1.75 mg integrated group.

The controlled trial did report treatment-emergent adverse events (TEAEs). The most common occurrences took place in the 1.25 and 1.75 mg groups. The TEAEs include nausea (22–24%), flushing (14–17%), and headache (9–14%). Seventy patients in the bremelanotide group and seven patients in the placebo group experienced injection-site reactions (irritation, rash, urticaria, swelling, pruritus, warmth, erythema, hematoma, hemorrhage, induration, nodule, or pain). Three bremelanotide subjects and one placebo subject reported serious TEAEs but were associated with underlying medical conditions instead of bremelanotide usage. 13 subjects withdrew from double-blind treatment due to associated TEAEs.

Overall, bremelanotide was fairly well tolerated. Of the patients who experienced injection-site reactions, 94% experienced mild events. Out of the 296 bremelanotide users and 98 placebo users, subjects in the bremelanotide group reported more frequent adverse reactions than placebo users for nausea (22% vs. 3%), vomiting (4% vs. 0%), and flushing (16% vs. 0%). Although BP recordings demonstrated a decline in mean BP (3–6 mmHg) and heart rate (5%), ABPM changes were not considered statistically significant between the bremelanotide and placebo groups.

From the previous phase 2b study, a responder analysis was carried out to evaluate the minimal clinically important difference (MCID) for patient-reported outcomes (PROs) [79]. Scores were selected from premenopausal women who were affected by HSDD or mixed HSDD/FSAD. Responder analyses evaluated the change from baseline to end-of-study for seven endpoints. Each PRO endpoint used 1–4 different types of responder analyses: planned analysis based on expert estimates, post hoc analyses, receiver operating characteristic (ROC) curves, and cumulative distribution function. From the responder rates, those at the 1.75 mg dose in the miTT population showed statistical significance compared with the placebo (*p* ≤ 0.03) at both primary and secondary endpoints. As a result, 1.75 mg bremelanotide was chosen by researchers for phase 3 clinical studies to further test efficacy and safety.

### 5.4. RECONNECT

Two-phase 3, randomized, double-blind, placebo-controlled, multicenter clinical trials (RECONNECT) were conducted between 2015–2016 to evaluate the safety and efficacy of bremelanotide treatment for premenopausal women with HSDD [78]. Premenopausal women 18 years or older with a stable monogamous relationship were recruited for the studies. HSDD diagnosis was determined using the Diagnostic Screening Guide for HSDD by an investigator or licensed health care provider. Subject eligibility was based on a diagnosis of HSDD for more than six months and evidence of normal sexual functioning for at least two years. Women who were pregnant, nursing, diagnosed with other female sexual dysfunction disorders, or currently being treated for any psychiatric disorders were excluded. Based on the data from a previous phase 2b clinical trial, 1.75 mg bremelanotide was selected for testing due to optimal efficacy and safety profiles.

Women randomized to the safety (*n* = 1247) and efficacy (*n* = 1202) groups received either the treatment or placebo for 24 weeks. Safety evaluations were performed at screening, monthly, then at the end of the study. Components included a physical examination, BP assessment, ECG, and clinical laboratory testing. Primary efficacy endpoints involved a change in Female Sexual Function Index-desire domain (FSFI-D) and Female Sexual Distress Scale-Desire/Arousal/Orgasm (FSDS-DAO) score from baseline to end-of-study. Secondary endpoints measured the change in satisfying sexual events within 16 h of receiving treatment or placebo and reported within 72 h. Because both studies were identical in design, based on exclusion/inclusion criteria, and efficacy and safety assessments, results collected from each study were integrated together in this review.

In these two phase 3 trials, it was demonstrated that bremelanotide showed clinical significance in the increase in sexual desire and decrease in distress related to low sexual desire compared with placebo for premenopausal women with HSDD. Both primary endpoints were achieved in the phase 3 trials. Specifically, women who took bremelanotide had a statistically significant increase in sexual desire for each independent study and combined (study 301: 0.30, *p* < 0.001; study 302: 0.42, *p* < 0.001; integrated studies 0.35, *p* < 0.001) while having a significant decrease in distress associated with low sexual desire (study 301: −0.37, *p* < 0.001; study 302: −0.29, *p* = 0.005; integrated studies −0.33, *p* < 0.001). The co-primary endpoints not only yielded statistically significant data between treatment groups but also had a significant response rate for studies 301 and 302 (bremelanotide: 58.3 and 58.2%, placebo: 36.1% and 35.4%). Higher response rates for the bremelanotide group signified that the subjects found more meaningful clinical changes when administered the treatment.

In both studies from baseline to the end of the study, women who were taking bremelanotide had a statistically significant increase in sexual desire (study 301 0.30, *p* < 0.001, study 302 0.42, *p* < 0.001, integrated studies 0.35, *p* < 0.001) and had a statistically significant reduction in distress related to low sexual desire (study 301 −0.37, *p* < 0.001, study 302 −0.29, *p* = 0.005, integrated studies −0.33, *p* < 0.001) compared with the placebo [81]. The secondary endpoint was the change from baseline to end-of-study in the number of satisfying sexual events that occurred within 16 h of study drug doses and reported within 72 h of occurring [81]. The change between the baseline and end-of-study number of satisfying sexual events reported did not reach statistical significance between the treatment groups (study 301 bremelanotide 0.0, placebo −0.1 *p* = 0.764, study 302 bremelanotide 0.0, placebo 0.0 *p* = 0.702, integrated studies bremelanotide 0.0, placebo −0.1 *p* = 0.630). The authors performed a post hoc exploratory analysis that showed that the difference in the percentages of sexual events that were considered satisfying sexual events (defined as the number of satisfying sexual events per total number of sexual encounters) increased in the bremelanotide group when compared to the placebo group. Across both studies, the differences in the percentage increased greater than two-fold in the bremelanotide group when compared to the placebo (25% vs. 9.8% *p* < 0.001) [81].

Subjects who received bremelanotide experienced more adverse side effects compared to those who received the placebo (76.6% vs. 58.2%). In both studies, bremelanotide users experienced more frequent nausea (40% vs 1.3%), flushing (20.3% vs 0.3%), and headache (11.3% vs 1.9%) than the placebo group. The TEAEs related to bremelanotide were nausea, flushing, and headache. As reflected in the original phase, 2b study, mean increases in subject BP and HR were modest. Of the 40% of the patients taking bremelanotide, only 8.1% discontinued the medication during the study due to this TEAE [81]. There were no clinically significant changes to the other safety measures evaluated.

Overall, the phase 3 trials have shown that Bremelanotide holds promise as a treatment for HSDD in postmenopausal women. The drug has demonstrated a clinical efficacy profile with minimal safety concerns, which ultimately led to FDA approval in 2019 [67]. The TEAEs experienced were related to tolerability and not safety concerns. It is important to note, however, that the high response rates of the RECONNECT trial could be attributed to the placebo effect, and this was noted by the authors. Table 1 summarizes the studies discussed in this section.

## 6. Conclusions

Hypoactive sexual desire disorder is a multi-faceted disorder involving biological, psychological, and pharmacological influences. The sensitivity of this disorder often makes it difficult to identify and treat. Further complicating treatment is the complex intertwinement between the biological and psychosocial causes of HSDD. Psychosocial factors such as relationship status, culture, and menopausal status have been shown to influence sexual desire and activity in women [10]. Additionally, the activity of hormones such as dopamine, testosterone, and progesterone have been shown to have positive effects on sexual desire, while other hormones such as serotonin and prolactin have been shown to impair sexual desire by inhibiting dopamine release [26,27]. Treatment of HSDD is focused on reducing sexual distress and improving sexual desire. Treatment options for HSDD include both psychosocial approaches and pharmacotherapy. Psychosocial treatments include cognitive behavioral therapy, mindfulness-based therapy, and couple’s therapy. Pharmacotherapy options include flibanserin, bupropion, ospemifene, testosterone, and bremelanotide [2]. Bremelanotide is the most recent FDA-approved treatment for HSDD. Bremelanotide is a nonselective melanotropin receptor agonist which primarily acts on MC4R receptors to modulate brain pathways involved in sexual responses [81]. Bremelanotide has shown efficacy in improving sexual responses in women suffering from HSDD. In comparison to a placebo group, women taking bremelanotide have reported increased sexual desire and more positive responses to feelings of genital arousal. Additionally, women taking bremelanotide reported increased satisfaction with arousal levels when attempting intercourse [76]. Bremelanotide should be used cautiously in patients with cardiovascular disease as it can decrease heart rate and increase blood pressure [68]. The most common side effects of bremelanotide are nausea, vomiting, and flushing [69]. Bremelanotide shows a promising way to treat HSDD.

## Figures and Tables

**Table 1:** Clinical Efficacy and Safety.

Author (Year).	Groups Studied and Intervention	Results and Findings
Diamond LE et al., 2006	Evaluated physiological and subjective measurements of sexual arousal and desire in premenopausal women diagnosed with HSDD (*n* = 18). Intranasal doses of bremelanotide or matching placebo on the first and second in-clinic visit. In each session, women were shown a 20-min normal video followed by a 20-min sexually explicit video. Perceived sexual response evaluated using questionnaires and vaginal vasocongestion (measured by vaginal photoplethysmography).	When administered bremelanotide women reported moderate or high sexual desire compared to placebo (*p* = 0.0114). Women reported more positive responses to feelings of genital arousal (*p* = 0.0833), and more satisfaction with level of sexual arousal when attempting sexual intercourse in the following 24 h (*p* = 0.0256). No adverse effects from BMT administration.
Clayton AH et al., 2017	Phase I Evaluated the safety, tolerability, and hemodynamic and pharmacokinetic effects of bremelanotide (BMT) when administered with ethanol. 24 participants (12 men; 12 women) enrolled in the study; received BMT or placebo with or without ethanol for 7 consecutive days. Participants received single intranasal doses of 20 mg BMT or placebo on days 1, 4, and 7, with or without oral ethanol (0.6 g/kg).	Doses of 20 mg intranasal BMT, with or without 0.6 g/kg ethanol, were considered safe and well tolerated with. No clinically significant pharmacokinetic interactions found between ethanol and BMT (both overall and by sex). No significant drug-related hypotensive or orthostatic hypotensive effects were noted. Unchanged frequency of treatment-emergent adverse events (TEAEs), no participants discontinued the study because of adverse events. Physical examination, electrocardiography, and laboratory tests disclosed no clinically significant changes.
Clayton AH et al., 2016	Phase 2b Premenopausal nonpregnant women at least 21 years old with FSAD, HSDD or a combination of these disorders for at least the preceding 6 months. Patients randomized to receive placebo or BMT 0.75, 1.25 or 1.75 mg self-administered subcutaneously over 12 weeks. Double-blind treatment began with two self-administered in-clinic study-drug doses spaced approximately 1 week apart. Dosing at-home, as-desired approximately 45 min prior to anticipated sexual activity. Primary end point was change in satisfying sexual events/month. Secondary end points included total score changes on female sexual function index and female sexual distress scale-desire/arousal/orgasm.	The primary efficacy end point was each patient’s change, from baseline to end of study (EOS), in the number of SSEs. The mean change in number of SSEs from baseline to EOS was +0.7 (2.4) events/month for BMT 1.25/1.75 mg pooled, compared with +0.2 (2.3) for placebo (*p* = 0.0180) Secondary end points included change from baseline to EOS in total FSFI score, FSFI domain scores for desire and arousal, total FSDS-DAO score and individual FSDS-DAO item #14 (arousal) and #13 (desire) scores. Mean change in FSFI total score was +3.6 (5.7) versus +1.9 (5.9; *p* = 0.0017) and the mean change in FSDS-DAO total score was −11.1 (12.0) versus −6.8 (13.6; *p* = 0.0014)
Kingsberg AS et al., 2019	RECONNECT Evaluated the safety and efficacy of bremelanotide 1.75 mg administered. For premenopausal women with hypoactive sexual desire disorder. Randomized 1:1 to 24 weeks of treatment with bremelanotide or placebo. Primary end point was change in FSFI-D and FSDS-DAO. Secondary end point was the number of satisfying sexual events.	Women who were taking bremelanotide had a statistically significant increase in sexual desire (study 301 0.30, *p* < 0.001, study 302 0.42, *p* < 0.001, integrated studies 0.35, *p* < 0.001). Statistically significant reduction in distress related to low sexual desire (study 301 −0.37, *p* < 0.001, study 302 −0.29, *p* = 0.005, integrated studies −0.33, *p* < 0.001) compared with placebo. Patients taking bremelanotide experienced more nausea, flushing, and headache (10% or more in both studies) compared with placebo.

## Data Availability

All data and materials found in this manuscript can be found in articles found on pubmed.
